# Stem cell therapy in diabetic men with erectile dysfunction: a 24-month follow-up of safety and efficacy of two intracavernous autologous bone marrow derived mesenchymal stem cells injections, an open label phase 2 clinical trial

**DOI:** 10.1186/s12610-024-00229-y

**Published:** 2024-07-05

**Authors:** Saddam Al Demour, Sofia Adwan, Hanan Jafar, Hussam Alhawari, Abdalla Awidi

**Affiliations:** 1 Department of Special Surgery, Division of Urology, School of Medicine, The University of Jordan, Amman, 11942 Jordan; 2 Dr. Sulaiman Al Habib Medical Group, Riyadh, Kingdom of Saudi Arabia; 3 Cell Therapy Center, The University of Jordan, 11942 Amman, Jordan; 4 Department of Medical Laboratories, Faculty of Health Sciences, American University of Madaba, 11821 Madaba, Jordan; 5 Department of Internal Medicine, School of Medicine, The University of Jordan, 11942 Amman, Jordan; 6 Department of Hematology-Oncology, Jordan University Hospital, The University of Jordan, Amman, 11942 Jordan

**Keywords:** Erectile dysfunction, Diabetic erectile dysfunction, Stem cells, Bone marrow-derived mesenchymal stem cells, Stem cells therapy, Dysfonction érectile, Dysfonction érectile diabétique, Cellules souches, Cellules souches mésenchymateuses dérivées de la Moelle osseuse, Thérapie par Cellules souches

## Abstract

**Background:**

Recently we reported results of phase 1 pilot clinical trial of 2 consecutive intracavernous (IC) injection of autologous bone marrow-derived mesenchymal stem cells (BM-MSCs) for the first time in the treatment of diabetic patients with erectile dysfunction (DM-ED). In phase 2 of this study our aim is to evaluate long term safety and efficacy of IC injections of BM-MSC on additional eight patients with DM-ED.

**Results:**

Each patient received 2 consecutive IC injections of BM-MSC and evaluated at 1, 3, 6, 12, and 24-month time points. Primary outcome was the tolerability and safety of stem cells therapy (SCT), while the secondary outcome was improvement of erectile function (EF) as assessed using the International Index of Erectile Function-5 (IIEF-5), Erection Hardness Score (EHS) questionnaires, and Color Duplex Doppler Ultrasound (CDDU). IC injections of BM-MSCs was safe and well-tolerated. Minor local and short-term adverse events related to the bone marrow aspiration and IC injections were observed and treated conservatively. There were significant improvement in mean IIEF-5, EHS, all over the follow-up time points in comparison to the baseline. At 24-month follow up there were significant decline in the mean IIEF-5, and EHS compared to the baseline. The mean basal and 20-min peak systolic velocity was significantly higher at 3-month after the IC injections compared to baseline.

**Conclusions:**

This phase 2 clinical trial confirmed that IC injections of BM-MSC are safe and improve EF. The decline in EF over time suggests a need for assessing repeated injections.

**Clinical trial registration:**

NCT02945462

## Résumé

**Contexte:**

Récemment, nous avons rapporté les résultats d’un essai clinique pilote de phase 1, de 2 injections intracaverneuses (IC) consécutives de cellules souches mésenchymateuses autologues dérivées de la moelle osseuse (BM-MSC), pour la première fois dans le traitement de patients diabétiques atteints de dysfonction érectile (DM-ED). Dans la phase 2 de cette étude, notre objectif est d’évaluer l’innocuité et l’efficacité à long terme des injections IC de BM-MSC sur huit autres patients atteints de dysfonction érectile.

**Résultats:**

Chaque patient a reçu 2 injections IC consécutives de BM-MSC, et a été évalué à des intervalles de temps de 1, 3, 6, 12 et 24 mois. Le critère de jugement principal était la tolérance et l’innocuité de la thérapie par cellules souches, tandis que le critère de jugement secondaire était l’amélioration de la fonction érectile (FE) évaluée à l’aide de l’indice international de la fonction érectile-5 (IIEF-5), de questionnaires sur le score de dureté de l’érection (EHS) et de l’échographie Doppler duplex couleur. Les injections IC de BM-MSC se sont avérées sûres et ont été bien tolérées. Des effets indésirables locaux et à court terme mineurs, liés à l’aspiration de la moelle osseuse et aux injections d’IC, ont été observés et traités de manière conservatrice. Il y a eu une amélioration significative des moyennes de l’IIEF-5 moyen, de l’EHS à tous les points de suivi par rapport à la l’état basal. A 24 mois de suivi, il y a eu une baisse significative de l’IIEF-5 moyen et de l’EHS par rapport à l’état basal. La moyenne se base et celle du pic maximal de la  vitesse systolique à 20 minutes étaient significativement plus élevées 3 mois après les injections de CI par rapport à l’état de base.

**Conclusions:**

Cet essai clinique de phase 2 a confirmé que les injections de BM-MSC par injections intracaverneuses sont sûres et améliorent la fonction érectile. La baisse de cette dernière au fil du temps suggère une nécessité d’évaluation des injections répétées.

## Introduction

Globally, erectile dysfunction (ED) is a major public health concern with estimated prevalence was 152 million in 1995 and predicted to increase to 322 million by 2025 [1]. Also, ED has a significant negative impact on quality of life of patients and their partners [2]. Diabetes mellitus (DM) is one of the most important risk factors for ED, and diabetic men exhibit a higher prevalence of ED than non-diabetic men with epidemiological studies documenting that up to 75% of diabetic men suffer from ED [3, 4]. Diabetic-ED (DM-ED) involves nerve damage, endothelial injury, and cavernosal muscle fibrotic alterations [5]. Unfortunately, only 50–60% of DM- ED patients can be successfully treated with phosphodiesterase type 5 inhibitor, as a first line treatment, thus making it essential to explore new therapeutic approaches [6].

Recently, stem cells (SCs) therapy is a highly promising novel treatment for DM-ED and have received much attention regarding their ability to regenerate damaged penile neurovascular and endothelial tissues [7]. Most of the evidence on SCs therapy (SCT) for ED was based on preclinical trials that reported encouraging results regarding improvements in functional and structural changes [8, 9, 10].

SCs are defined by their self-renewal capability and differentiation potential and classified as totipotent, pluripotent, or multipotent SCs [11]. Multipotent SCs, such as haematopoietic SCs and MSCs, are isolated from the developing germ layer and their descended adult organs, can renew themselves and differentiate into any cell type within their germ layer. MSCs can be harvested from a variety of sources, including bone marrow, adipose tissue, muscle tissue, urine, umbilical cord blood, and Wharton’s jelly [12].

Using MSCs in the treatment of DM-ED is shown to have therapeutic benefits not only because these cells are known to secrete various growth factors causing a stimulatory paracrine effect, but also because of their anti-inflammatory and angiogenic activities, as well as possibility of differentiating into tissue relevant to the penile architecture [13, 14, 15].

In contrast to the large number of animal studies in the treatment of ED, only a limited number of human studies were conducted to evaluate the safety and efficacy of autologous BM-MSCs in treatment of DM-ED [16, 17, 18]. To the best of our knowledge, this is one of the first human clinical studies using 2 consecutive IC injections of autologous BM-MSCs in the treatment of DM-ED. In this phase 2 pilot study, we aimed to investigate the long-term safety of this new promising therapeutic approach as a primary outcome, and efficacy as a secondary outcome.

## Methods

### Study Design and approval

This is an open-label, single-center, phase 2 pilot clinical trial designed to evaluate the 24-month safety and potential efficacy of autologous BM-MSCs therapy in DM-ED patients. The study was approved by the institutional review board at Cell Therapy Center/ The University of Jordan and prospectively registered on clinicaltrials gov (NCT02945462). The study protocol complied with the Declaration of Helsinki. Written informed consent was obtained from all patients before study enrolment.

### Inclusion and exclusion criteria

Participants were enrolled in the study if they met all the eligibility criteria outlined in (Table 1).

**Table 1:** Eligibility criteria for patients

Inclusion criteria
Age ranging from 25 to 65 years.
Type 1 or type 2 diabetes with an HbA1c ≤ 10%.
History of diabetes ≥ 5 years.
Body mass index between 20 and 30.
Baseline International Index of erectile function (IIEF-5) score of < 22.
History of chronic erectile dysfunction for at least 6 months.
Exclusion criteria
penile anatomical deformities (e.g., Peyronie’s disease).
Penile skin irritation, infection, or wound in the immediate areas of skin entry for penile injection.
Bleeding or clotting disorders.
Current urinary tract infection, current or previous infection with human immuno- deficiency or hepatitis viruses.
Previous penile implant, penile vascular surgery, or radical prostatectomy.
Current or previous malignancy.
Prostate-Specific Antigen (PSA) (> 4 ng/mL).
Untreated hypogonadism or low serum total testosterone (< 200 ng/dL).
Uncontrolled hypertension or hypotension (systolic blood pressure > 170 or < 90 mm Hg, and diastolic blood pressure > 100 or < 50 mm Hg).
Cardiovascular disease (e.g., unstable angina, myocardial infarction within past 6 months, cardiac failure or life-threatening arrhythmia, and congestive heart failure) or symptomatic postural hypotension within 6 months before screening.
Systemic autoimmune disorder.
The following laboratory screening results also had to be normal: luteinizing hormone, testosterone and prolactin, liver function tests (ALT, AST, and GGT), kidney function tests and/or electrolytes (urea, creatinine, Na, K, and Ca), complete blood count with differential, coagulation profile (INR, PT, and PTT), lipid profile (HDL, LDL, TG, and total cholesterol), urinalysis and culture, hepatitis B and C (HBs Ag and hepatitis C antibodies), human immune deficiency, and lues serology (VDRL).

### Isolation, culture and expansion

Bone marrow (BM) isolation and preparation were performed as previously described by our group [16, 19]. Briefly, autologous bone marrow aspiration was performed under local anesthesia, and mononuclear cells were isolated from the BM aspirates using Histopaque-1077 (Sigma-Aldrich) density gradient centrifugation. Cells were seeded at a density of 1.6 × 10^5^ cells/cm^2^ in complete alpha-MEM containing 10% human platelet lysate, 4 mML-glutamine (Gibco), and 100 U/mL penicillin/streptomycin (Gibco). Following 24 h, non-adherent cells were removed and adherent cells were maintained in growth culture medium for 10–14 days, and was replaced twice weekly. Cells were detached at 70–80% confluence with TrpLE 10X (Gibco), and plated at a seeding density of 4,000 cells/cm^2^ for subculture. Cells were then cryopreserved in synth-a-freeze freezing media (Gibco) at their first passage to be used for the preparation of the second injection. Second and third passages were used for the injections, characterization and release tests. All cell culture and preparation were performed under current good manufacturing practice (cGMP) guidelines.

### Mesenchymal stem cells characterization

Expanded MSCs were characterized in accordance with main criteria defined by the international society for cellular therapy (ISCT) [20], such as their multilineage differentiation capability toward the adipogenic and osteogenic lineages and their expression of MSCs surface markers. Surface markers were tested using BD Stemflow™ hMSC Analysis kit and their fluorescence intensity was evaluated by flow cytometry (BD FACS canto II, BD biosciences). Furthermore, the osteogenic and adipogenic differentiation potential was assessed by StemPro® Ostogenesis Differentiation and StemPro® Adipogenesis Differentiation Kits (Gibco), respectively as per manufacturer’s instructions. Following induction, osteogenic differentiation was demonstrated by mineral deposition detected through Alizarin red S staining (Allied Signal) whereas adipogenic differentiation was confirmed by the accumulation of lipid vacuoles and oil red O staining (Sigma-Aldrich).

### MSC release tests

The following criteria were set as release criteria for the characterized viable MSCs: The absence of mycoplasma contamination, sterility of bacterial and fungal growth, and free of endotoxin contamination. Cell viability was assessed through two trypan blue exclusion-based methods: Manually using hematocytometer and using Countess automated cell counter (Thermo, USA). Percentages of cell viability ≥ 80% were considered acceptable. Patients’ cells and culture medium were screened and tested for mycoplasma contamination using MycoSEQ™ Mycoplasma Detection Kit (Invitrogen) and were performed according to the manufacturer’s recommendations. Additionally, the culture medium was cultured on blood, MacConkey, and chocolate agar plates (in an anaerobic jar) at 37 °C for 72 h. And finally, Limulus amebocyte lysate QCL-1000 (Lonza, Switzerland) test was used to detect Gram-negative bacterial endotoxin. The limulus amebocyte lysate test was performed according to the manufacturer’s instructions [21].

### Patient Assessment and Clinical Procedure

At baseline, patients were requested to fill in validated IIEF-5 and Erection Hardness Score (EHS) questionnaires. All patients underwent a thorough medical and sexual history, complete physical examination, and laboratory investigations.

Eligible patients underwent penile CCDU as baseline assessment of penile hemodynamics by measuring peak systolic velocity (PSV), end-diastolic velocity (EDV), both measured in cen- timeter per second, and the resistive index (RI) in both cavernosal vessels before (basal PSV, EDV, and RI) and 20 min after an IC injection of 20 µg of alprostadil (20-min PSV, 20-min EDV, and 20-min RI). After that, each eligible patient received two doses (IC injection) of ex-vivo expanded autologous BM-MSCs, with a 30-day interval between both. Each time, passage 2 or passage 3 cells were used for the injections; these were suspended at a density of 20 × 10^6^ cells/4 mL normal saline and loaded into 1 mL sterile syringes. At each time IC injections were given at 4 sites: 1 proximal and 1 distal injection into each corpus cavernosum. Patients were instructed not to take any medications to treat ED for the study’s whole duration.

### Outcome measures

Tolerability was assessed by observing and questioning patients for pain during IC injections; pain intensity was assessed on a 0–10 visual analog scale (VAS), in which 0–3 represented mild pain, 4–6 represented moderate pain, and 7–10 represented severe pain. Safety outcomes were assessed immediately, at 24 h, 1, 3, 6, 12, and 24-month after IC injections.

Safety was assessed by examination of the injection sites for bleeding, bruising, tenderness, swelling, erthermia, urticaria, or indurations, by measuring the vital signs and by interviewing patients for any other potential adverse events that might have occurred. Specifically, patients were asked at visit about nervous system symptoms, cardiovascular symptoms, respiratory symptoms, gastro-intestinal symptoms, and urinary tract symptoms. Moreover, the same laboratory tests taken at baseline were repeated at 1 and 3 months during follow-up.

Efficacy of the IC injections of autologous BM-MSCs was assessed qualitatively by IIEF-5 and EHS questionnaires at 1, 3, 6, 12, and 24-month of follow-up. Moreover, the follow-up with penile CDDU was performed 3 months after the second IC injection.

### Statistical analysis

This was a pilot study; thus, no sample size calculations were conducted. Descriptive data were reported as mean ± SD. Efficacy outcomes were compared at each point of the follow-up with baseline using Wilcoxon signed-rank test. A statistical signifiacance level of 5% and a 2-tailed test were used. Statistical analyses were performed using IBM SPSS Statistics for Windows, version20 (IBM Corp., Armonk, NY, USA).

## Results

### Patient Population

**Fig. 1:**
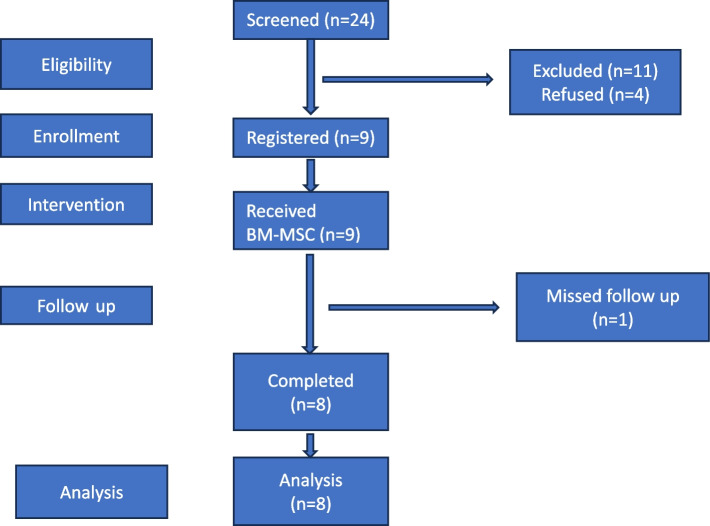
Flowchart of patients during the study period

A total of 24 patients were enrolled in this phase 2 clinical trial. Eleven patients were excluded from the study as they did not meet the inclusion criteria, four patients refused to participate, and 1 patient missed follow-up. A total of 8 patients underwent final analysis (Fig. 1). The mean age of patients was 55 ± 6.8 years. A summary of patients’ demographics is presented in (Table 2).

**Table 2:** Patients demographics (*n* = 8)

**Age (years)** **Mean ± SD**	55 ± 6.8
**BMI (kg/m**^**2**^**)** **Mean ± SD**	26.2 ±2.5
**DM duration (years)** **Mean ± SD**	10.9±3.7
**Smoking (n) (%)**	6 (75)
**Co-morbidity (n) (%)**	(5) (62.5)

*BMI* body mass index, *DM* diabetes mellitus, *SD* standard deviation

Data presented as mean ± SD; or frequency (percentage)

### Characterization and release of BM-MSCs

**Fig. 2:**
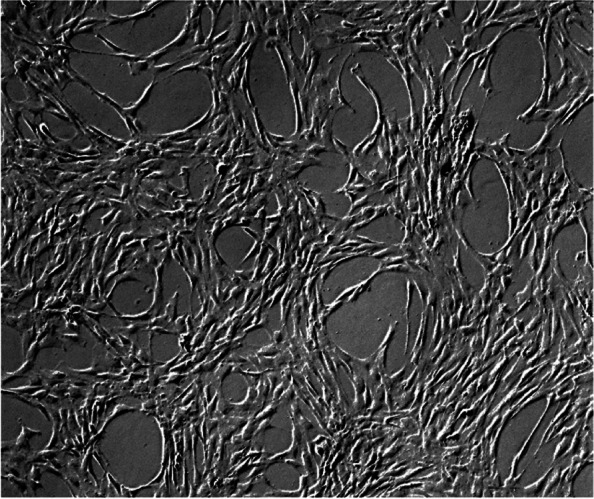
BM- MSCs. Representative light microscopy image of BM-MSCs at P2 before injection. BM, Bone marrow; MSCs, mesenchymal stem cells

**Fig. 3:**
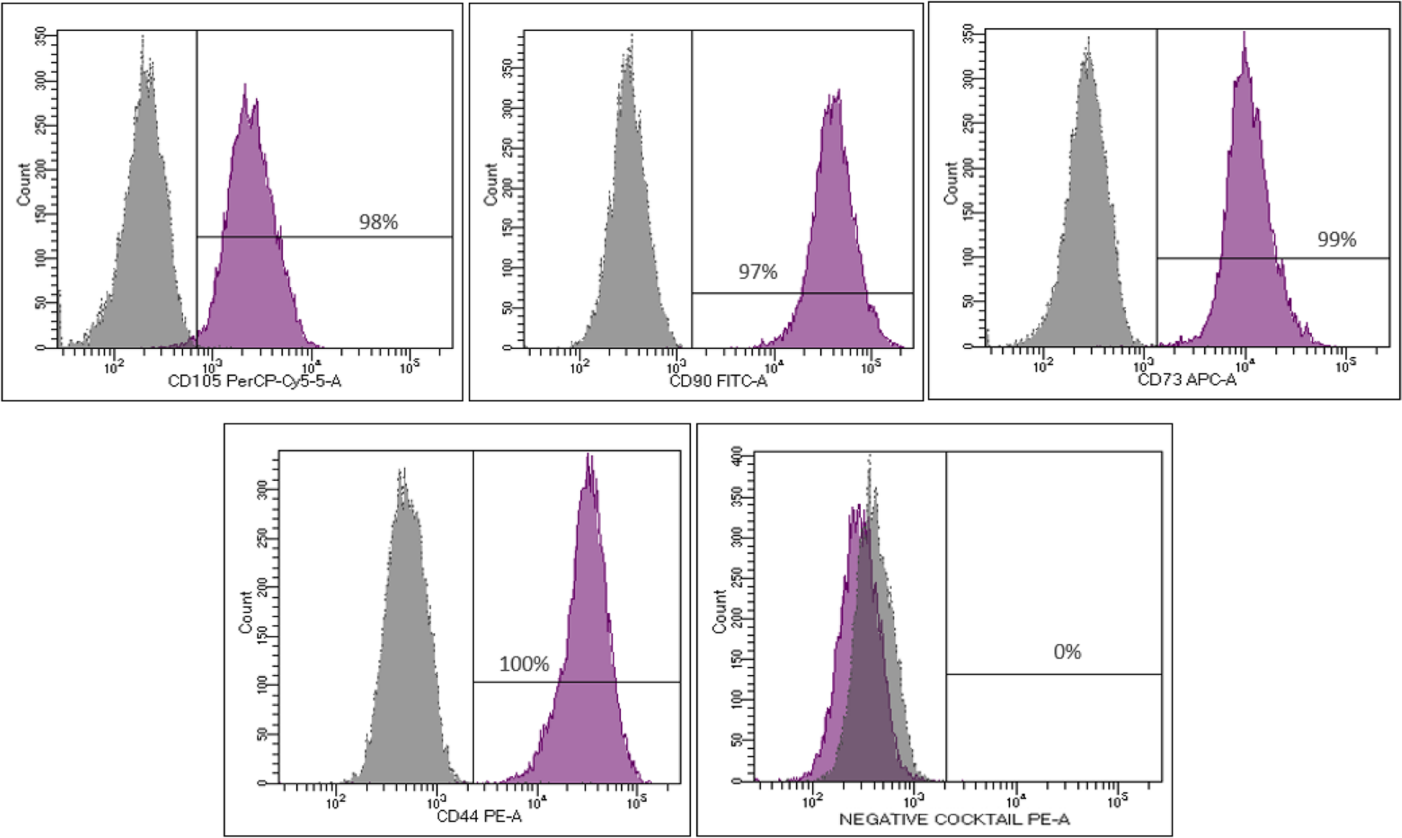
Flow cytometric characterization of BM-MSCs. Positive expression of CD90, CD105, CD73, and CD44, and negative expression of hematopoietic markers: CD34, CD45, CD11b, CD19, and HLA-DR. MSCs, mesenchymal stem cells

**Fig. 4:**
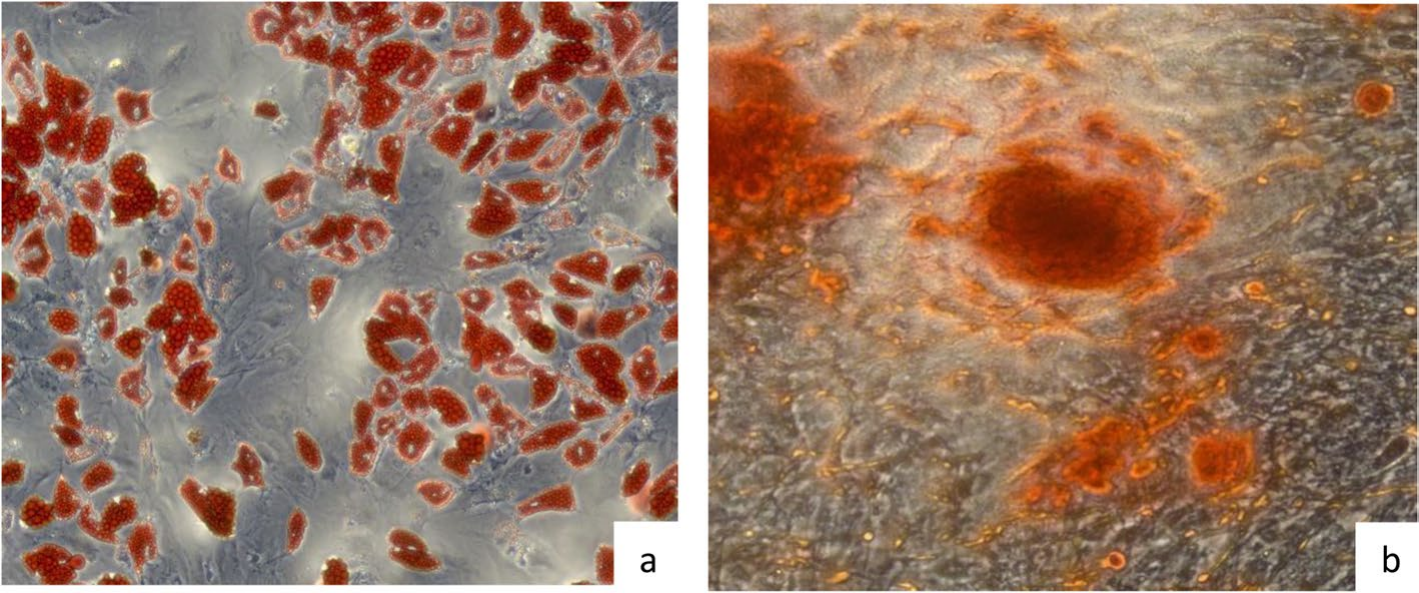
Representative light microscopy images (40x) of the in vitro differentiation of BM-MSCs toward adipogenic and osteogenic lineages (**a**) Adipogenic differentiation of BM-MSCs stained with Oil Red O. **b** Osteogenic differentiation of BM-MSCs stained with Alizarin red. BM, Bone marrow; MSCs, mesenchymal stem cells

The released patients’ BM-MSCs grew in a spindle shape which is a typical fibroblast-like cell morphology (Fig. 2). These were also positive for MSCs signature markers determined by ISCT; CD90, CD105, CD73 and CD44 and were negative for CD34, CD45, CD11b, CD19 and HLA-DR (Fig. 3). Moreover, upon differentiation induction, patients’ BM-MSCs were able to differentiate toward adipogenic and osteogenic lineages (Fig. 4a and b, respectively). All cultures were free of mycoplasma, bacterial, and endotoxin contamination.

### Primary outcomes

The primary outcomes were safety and tolerability. The procedure was well-tolerated, safe, and no serious adverse effects were reported. Five patients reported moderate pain at bone marrow aspiration site only during the procedure; the VAS pain score ranged from 4 to 6, and relieved after 4 h of conservative management. Seven patients reported mild penile pain and redness at the injection site only at the time of the procedure; the VAS pain score ranged from 0 to 3. Three patients reported minimal swelling, and bruises on the penile shaft 6 h after injection which was resolved within 3 days of conservative treatment. On observing the injection sites during follow-up, the majority of patients had no pain, bleeding, erythema, urticaria, bruising, swelling, priapism, hypothermia, or hyperthermia. Vital signs (body temperature, heart rate, respiratory rate, and blood pressure) were normal in all patients at all follow-up visits. Additionally, there were no systemic adverse events detected during follow-up, including cardiovascular, respiratory, urinary, nervous, and gastrointestinal systems. Furthermore, standard biochemistry and hematology laboratory tests showed no abnormal results at 1-month after the first IC injections and 3-month after the second IC injections.

### Secondary outcome

**Fig. 5:**
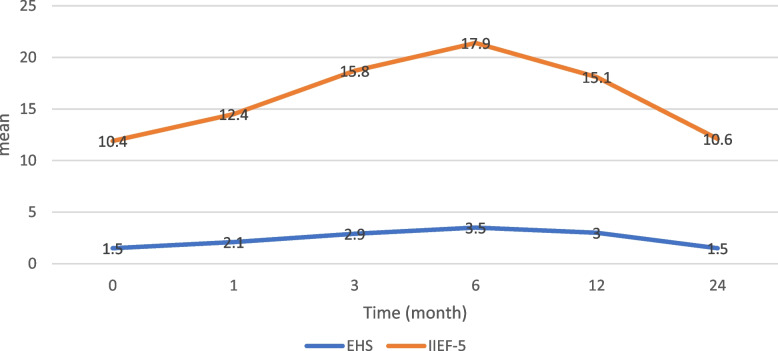
Changes in mean IIEF-5 and EHS scores after intracavernous injection BM-MS (*n* = 8). IIEF-5 range from 5 to 25 scores: severe (5-7), moderate (8-11), mild to moderate (12-16), mild (17-21), and no ED (22-25). EHS scale: 0, penis does not enlarge; 1, penis is larger but not hard; 2, penis is hard but not hard enough for penetration; 3, penis is hard enough for penetration but not completely hard; 4: penis is completely hard and fully rigid. Values are presented as mean ± standard deviation. The statistical analysis is performed via Wilcoxon signed rank test

Overall, the mean IIEF-5 and EHS scores were improved significantly at all follow-up time points except at 24-month compared to the baseline. There was a remarkable improvement of IIEF-5 score at 1-month after IC injections compared to baseline 12.4±2.6 vs. 10.4±2.8, (*p* = 0.0156). Also, 3-month after the injections, there was a further increase in scores with significant difference compared to the baseline scores 15.8±3.4 vs. 10.4±2.8, (*p* = 0.0078). After 6-month, the IIEF-5 scores reached maximum improvement compared to baseline 17.9±3 vs. 10.4±2.8, (*p* = 0.0003). At 12-month there was significant deference compared to baseline score 15.1±4.3 vs. 10.4±2.8, (*p* = 0.0168). However, at 12-month, there was a significant decline in the scores compared to the 6-month values 15.1±4.3 vs. 17.9±3, *p* = 0.0313. After 24-month the mean of IIEF-5 scores were declined with no significant difference compared to baseline 10.6±2.92 vs. 10.4±2.8, (*p* = 0.3506) (Table 3) (Fig. 5).

**Table 3:** Changes in mean of IIEF-5 score and EHS score after intracavernous injection of BM-MSC over follow up time points

	Baseline Mean±SD (*n* = 8)	1-month Mean±SD (*n* = 8)	*P*-value	3-month Mean±SD (*n* = 8)	*P*-value	6-month Mean±SD (*n* = 8)	*P*-value	12-month Mean±SD (*n* = 8)	*p*-value	24-month Mean±SD (*n* = 8)	*P*-value
**IIEF-5**	10.4±2.8	12.4±2.6	**0.0156**	15.8±3.4	**0.0078**	17.9±3	**0.0003**	15.1±4.3	**0.0168**	10.6±2.92	0.3506
**EHS**	1.5±0.5	2.1±0.6	**0.0492**	2.9±0.6	**0.0012**	3.5±0.5	**< 0.0001**	3±0.7	**0.0025**	1.5±0.53	0.5

*IIEF-5* International Index Erectile Function score, *EHS* Erection Hardness score, *SD* standard deviation

Values are presented as mean ± standard deviation. The statistical analysis is performed via Wilcoxon signed rank test

The EHS score also significantly improved over time points. At baseline, the mean score was 1.5±0.5 and it reached the maximum score at 6-month after IC injections 3.5±0.5, (*p* < 0.0001). There was also a significant drop in the mean score at 12-month compared to the 6-month value 3±0.7 vs. 3.5±0.5, (*P* = 0.0331) but was still higher than baseline value. At 24-month the mean of EHS score was declined to baseline level with no significant difference compared to baseline 1.5±0.53 vs. 1.5±0.5, (*p* = 0.5) (Table 3) (Fig. 5).

**Fig. 6:**
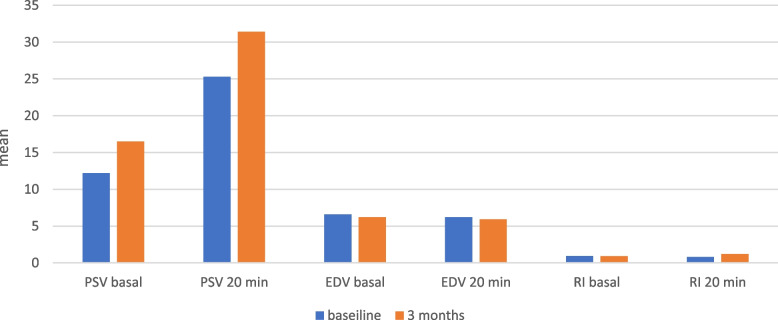
Color Duplex Doppler Ultrasound of the penis (CDDU) before and after BM-MSC injections (*n* = 8). Peak systolic velocity (PSV), end-diastolic velocity (EDV), and the resistive index (RI), basal: before IC injection of 20 μg of alprostadil injection, 20 min : 20 min after IC injection of 20 μg of alprostadil injection. Values are presented as mean ± standard deviation. The statistical analysis is performed via Wilcoxon signed rank test

The mean basal and 20-min PSV was significantly higher at 3 months after the IC injections compared to baseline 16.5±3 vs. 12.25±2.5, (*p* = 0.0039), 31.4±6.3 vs. 25.3±3.7, (*p* = 0.0055) respectively. The overall changes in the mean basal and 20-min of EDV and RI at baseline and 3-month after IC injection were not statistically significant (Table 4) (Fig. 6).

**Table 4:** Color Duplex Doppler Utrasound of the penis (CDDU) before and after BM-MSC injections

	Baseline (before BM-MSC injection) (*n* = 8)	3-month (after second BM-MSC injection) (*n* = 8)	*P*-Value
**Basal PSV** **Mean±SD**	12.25±2.5	16.5±3	0.0039
**Basal EDV** **Mean±SD**	6.6±2.4	6.2±2.7	0.1250
**Basal RI** **Mean±SD**	0.95±0.09	0.92±0.1	0.1250
**20 min PSV** **Mean±SD**	25.3±3.7	31.4±6.3	**0.0055**
**20 min EDV** **Mean±SD**	6.2±2.5	5.9± 2.6	0.3750
**20 min RI** **Mean±SD**	0.8±0.3	1.2±0.9	0.8146

Changes in meqan color duplex Doppler ultrasound parameters after the intracavernous injection of BM-MSC. We assessed penile vascularization by measuring peak systolic velocity (PSV), end-diastolic velocity (EDV), and the resistive index (RI) in both cavernosal arteries before (basal PSV, basal EDV, basal RI) and 20 min after an intracavernous (IC) injection of 20 μg of alprostadil (20-min PSV; 20 min-EDV; 20-min RI). Significant differences versus baseline are in bold type. Values are presented as mean ± standard deviation. The statistical analysis is performed via Wilcoxon signed rank test

*PSV* peak systolic velocity, *EDV* end-diastolic velocity, *RI* resistive index, *BM-MSCs* bone marrow mesenchymal stem cells, *IC* intracavernous, *SD* standard deviation, *CDDU* Penile Color Duplex Doppler Ultrasound, basal: before IC injection of 20 µg of alprostadil injection, 20 min : 20 min after IC injection of 20 µg of alprostadil injection

## Discussion

In recent years much attention has been given to SCT and few clinical trials have been conducted to evaluate the safety and efficacy in the treatment of DM-ED patients [16, 17, 18, 21, 22, 23, 24, 25, 26, 27].

Recently, we reported our results of phase 1, pilot clinical trial over 12-month follows up time, investigating the safety and potential therapeutic effect of 2 consecutive IC injection of autologous BM-MSCs in four DM-ED patients (NCT02945462) [16]. Here, in this phase 2 study we reported the final results of 24-month follow up of safety and efficacy after 2 consecutive IC injections of autologous BM-MSCs on additional eight DM-ED patients. We demonstrated that this intervention is tolerable and safe clinical approach over 24-month of follow up. Also, we reported remarkable clinical improvement on EF confirmed by significant changes on IIEF-5, EHS and PSV compared to baseline. At 24-month follow up point we reported significant decline in mean IIEF-5 and EHS compared to baseline. Our results are in the line with those reported by other researchers who have also shown SCT to be safe and indicating improvement of EF [18, 23, 25].

Several clinical trials investigated the safety of different types of SCs in the treatment of ED, reported no serious adverse events [18, 21, 23, 25, 26]. In our study no serious adverse events related to the intervention has been noted, only minor local and short-term adverse events related to the bone marrow aspiration and IC injections were observed and treated conservatively. Similarly, You et al. [26], conducted a stage 1 clinical trial assessed safety and efficacy of autologous BM-SCs in 10 patients with ED following radical prostatectomy ED (RP-ED) or DM-ED. One patient experienced two emergent adverse events (pyrexia and back pain), and two patients experienced a total of five adverse events (one case each of viral upper respiratory tract infection, prostatitis, and pruritus and two cases of hyperglycemia). One patient experienced two serious adverse events (two instances of hyperglycemia). Authors claimed that all adverse events were not related to autologous BM-SCs therapy. Also, Haahr et al. [25], reported that there were no serious adverse events occurred after single IC injection of autologous adipose-derived regenerative cells in patients with RP-ED, but eight reversible minor adverse events including transient redness, swelling, abdominal hematomas were observed.

Although, the exact mechanism of improvement, remains unclear [28], SCs have certain distinct properties, make them a promising clinical approach for treatment of DM-ED. In our study, the EF improvements were associated with a significant PSV increase suggesting an angiogenic effect of BM-MNC injections. While some authors suggest engraftment and differentiation of SCs, other authors attribute improvement in EF likely due to the paracrine factors secreted by the SCs, namely cytoprotective, anti-fibrotic, and anti-apoptotic molecules [29].

In the last decade, several human clinical studies reported variable results regarding the efficacy of SCs in the treatment of DM-ED. In our study we observed significant increase in the mean IIEF-5 and EHS over the 1, 3, 6, 12-month time points, then decline in the scores in 24-month of follow up comparable to the level of baseline 10.6±2.92 vs. 10.4±2.8 (*p* = 0.3506), 1.5±0.53 vs. 1.5±0.5 (*p* = 0.5) respectively. We attributed these significant changes either due to loss of BM-MSCs function or the normal age-related decrease in the EF. Of note, our previous study [16], showed a similar pattern of clinical improvement. However, in this current study, there was a lightly better response. We believe this is because the cells were cultured for a shorter period of time to yield the lower dose used in this current study. These findings are to be confirmed in a larger scale clinical trial. Based on the above findings we suggest that repeat injections may be needed to optimize or maintain the treatment effects of SCs.

Yiou et al. [22, 23] evaluated the safety and efficacy of intracavernous autologous BM-MSCs for post-RP-ED. In the stage 1 of their study, 12 patients were divided into 4 groups and treated with escalating BM-MSCs doses, demonstrating the safety and efficacy of the treatment. In the stage 2 of the study, six additional patients with longer-term follow-up (mean, 62.1 months) were injected with the optimal dose, as indicated by the stage 1 (1⋅10^9^ BM-MNCs). Significant improvements in intercourse satisfaction and EF domains of IIEF-15 and EHS were noted at 6-month follow-up, and clinical benefit was sustained after one year, especially with the highest dose (1⋅10^9^ BM-MNCs). Authors reported nonsignificant decline in the IIEF-erectile function at the last follow-up, compared with the 1-year time point (15.3± 8.1 vs. 18.1 ± 7).

Furthermore, You D et al. [26] reported that IIEF-5 was improved compared to baseline at all time points but were not statistically significant except at the first month time point. Levy et al. [27] conducted a study using a placental matrix derived MSCs in 8 patients with ED. The authors reported that there were no significant changes on the mean of IIEF-5 score, and they suggested that it is unlikely that one injection of any substance would be able to restore EF completely, but this treatment may help maximize penile blood flow and improve EF.

Recently, Mirzaei et al. [17] reported their results on efficacy of single IC injection of (50–60 × 10^6^ cells) autologous MSCs extracted from oral mucosa on 10 patients with DM-ED. The authors reported a significant improvement in the IIEF-5 score in the intervention group compared to the control group over 6 months follow up points. However, there was no significant difference in PSV, EDV, and RI in both groups. In our study we used 2 IC injections which are more efficient than single dose and might be the cause of improvement in IIEF-5, EHS scores, and PSV.

Three human clinical trials using MSCs in the treatment of ED patients who underwent RP for prostate cancer [23, 25, 26]. The pathophysiology of ED post-RP is actually quite different than that involved in diabetic patients. Physical injury to the neurovascular bundle is the principle pathogenesis of ED post RP, this ultimately may result in corporeal fibrosis and veno-occlusive dysfunction [30]. On the other hand, DM-ED is mainly a functional disorder resulting from impaired NO production by endothelial cells [31]. Therefore, we included only diabetic patients with ED in our study population as different pathophysiology may result in different response to treatment.

In summary, to our knowledge this is one of the first human studies to evaluate the 24-month of safety and efficacy of 2 consecutive IC injections of autologous BM-MSC for the treatment of DM-ED. The safety and tolerability were evaluated clinically and by extensive laboratory examinations. The efficacy of the treatment was evaluated subjectively by validated questionnaires and objectively by CDDU. Thus, this treatment was found to be tolerable, safe, as well as effective in improving EF in DM-ED patients. Moreover, this study assessing the effect of SCs therapy at 24-month follow-up showed a subsequent decline in the IIEF-5 and EHS scores, indicated that the improvement in EF is time limited.

The authors acknowledge that the current study has some limitations. First, this study was unblinded and without a control group. Second, the small number of patients recruited in this study was principally driven by the low social acceptance of this new treatment modality.Third, the absence of radiology studies for long-term safety evaluation represents a notable constraint that warrants acknowledgment and future attention. However, as we have demonstrated the safety and efficacy of this treatment, we are recruiting a large number of patients with prolonged follow-up periods. Further studies for dose-finding and studies with double-blinding and a control group will have to be undertaken in order to further assess this treatment for DM-ED patients.

## Conclusion

The current findings in this phase 2 human clinical trial support the safety and efficacy profile of 2 consecutive IC injections of autologous BM-MSCs to treat MD-ED. The gradual decline of IIEF-5 and EHS score seen after 12-month of follow-up may indicate that the improvement in EF is time limited that may suggests a need for assessing repeated injections. The potential efficacy of autologous BMSC treatment in patients with ED needs to be confirmed by a large sample, randomized, placebo-controlled clinical trial.

## Data Availability

The datasets generated and analyzed during the current study are not publicly available due to privacy but are available from the corresponding authors on reasonable request.

## Abbreviations

BM: Bone marrow
BM-MSCs: Bone Marrow-Derived Mesenchymal Stem Cells
CDDU: Color Duplex Doppler Ultrasound
DM-ED: Diabetic Erectile Dysfunction
ED: Erectile Dysfunction
EF: Erectile Function
EHS: Erection Hardness Score
EDV: End-Diastolic Velocity
IIEF-5: International Index of Erectile Function-5
IC: Intracavernous
MSCs: Mesenchymal Stem Cells
SCs: Stem Cells
SCT: Stem Cells Therapy
PSV: Peak Systolic Velocity
RI: Resistive Index
VAS: Visual Analog Scale
